# Evaluation of a Web-based Information Platform for Youths on Mental Health During the COVID-19 Pandemic

**DOI:** 10.1007/s10578-022-01425-5

**Published:** 2022-10-20

**Authors:** Regine Primbs, Charlotte Elisabeth Piechaczek, Lucia Iglhaut, Patricia Grill, Lisa Feldmann, Gerd Schulte-Körne, Ellen Greimel

**Affiliations:** Department of Child and Adolescent Psychiatry, Psychosomatics and Psychotherapy, Hospital of the Ludwig-Maximilians-University (LMU) Munich, Nußbaumstr. 5, D-80336 Munich, Germany

**Keywords:** COVID-19, Self-management, Prevention, Knowledge, Web-based

## Abstract

During the corona pandemic, youths were confronted with substantial challenges and in urgent need of information on how to prevent mental health problems. This experimental pre-post-follow-up study examined the efficacy and reception of an innovative website (www.corona-und-du.info) containing evidence-based information for youths on how to overcome psychological distress during the pandemic. 34 youths aged 11–18 years were presented domains of the website in the laboratory. Standardized questionnaires were applied to assess changes in knowledge, the reception of the website, and changes in behavioral intention to seek psychological help. For all content domains, we found a significant knowledge gain with large effect sizes from pre to post, and from pre to follow-up. The reception of the website was very positive, and participants indicated increased intention to seek psychological support when needed. The results constitute an important basis for future attempts to promote mental health information in youths in times of crisis.

The online version contains supplementary material available at 10.1007/s10578-022-01425-5.

## Introduction

The spread of the Coronavirus Disease 2019 (COVID-19) has caused an ongoing worldwide pandemic with rapid and restrictive temporal countermeasures and far-reaching consequences, especially for youths and their families. Particularly at the beginning of the pandemic, the countermeasures, including social distancing, contact reductions, school closings, and quarantine have imposed high burdens on youths [1–5]. The arising circumstances were highly incongruent with important milestones during childhood and adolescence, like gaining independence, exploring identity, and the increased importance of the peer group and social interactions with peers [6, 7]. Findings from recent studies on the impact of the pandemic on mental health of youths during the first year of the crisis found that children and adolescents in countries all over the world felt highly burdened by the pandemic, experienced more mental health problems [8–11], and have higher levels of depression and anxiety compared to pre-pandemic times [12, 13]; for a meta-analysis see [14], for a recent review see [4]. Girls, older adolescents [14] and youths with psychological and/ or chronic somatic conditions were at a higher risk to experience poor mental health outcomes [4, 14]. Importantly, children with low socioeconomic status, migration backgrounds as well as those with limited living space were more severely affected by the increase in mental health problems [13, 15].

Most children and adolescents know only little about promoting mental health and preventing mental illnesses like depression [16, 17]. However, promoting this kind of information can have positive effects on children’s and adolescents´ well-being. Such an approach aims at enhancing resources, competencies, and strengths of children and adolescents in order to increase their autonomy over their mental health and to overcome adversity [18].

Today, children and adolescents grow up as digital natives. In western countries, nearly all children and adolescents between 12 and 19 years have daily access to a computer and/or smartphone [19–21]. In this respect, also online formats on mental health are gaining increasing importance [e.g., 22], which provide new ways to reach the target groups and to facilitate access to mental health offers [23, 24]. In recent years, a number of online information platforms on mental health for children, adolescents, and adults [e.g., 25, 26, 27] have been launched, which show high acceptance rates [28, 29]. However, particularly with regard to digital platforms for youths to increase knowledge on mental health promotion, there is a scarcity of offers that have been evaluated.

First evaluation studies with healthy adolescents on the effectiveness of information material (e.g. brochures) provide promising results regarding increased knowledge about depressive disorders even over a period of four weeks. Of note, these studies show that school type and sex influence the extent of knowledge gain over time [30, 31]. Specifically, female participants [31] and participants attending higher school types [30, 31] exhibited more pronounced knowledge enhancement over time than male participants or participants attending lower school types, respectively.

Apart from the aspect of knowledge gain, a few evaluation studies have shown that digital platforms on mental health promotion in children and adolescents increase behavioral intention in terms of heightened help seeking behavior. One example is an interactive web-based health magazine for youths [27], which promotes health literacy and aims at encouraging users to seek help in case of mental health problems. An evaluation study of this offer has demonstrated that users who had visited the website were more likely to seek help compared to those who had not visited the website [32]; for a similar result see [33].

To our knowledge, no study to date has provided evidence whether information platforms on mental health for children and adolescents during the COVID-19 pandemic are effective in enhancing knowledge as well as behavioral intention. Evidence on this aspect, however, is of high priority in light of increased mental health problems in youth in the current crisis [e.g., 8, 9, 10] and the urgent need of effective approaches to promote mental health information to this important target group.

### Current study

Using a pre-post-follow-up design, the current study aimed at evaluating an evidence-based information portal on mental health for children and adolescents during the corona pandemic (“Corona und Du”) [34]. This platform contains evidence-based information for children and adolescents as well as advice and supporting information on how to prevent and deal with psychological stress during the corona pandemic and is subdivided in relevant content domains. In more detail, the study aimed at investigating whether the consumption of mental health contents of the website leads to a growth in knowledge in these content areas and whether this growth in knowledge is sustained at follow-up. Connected to this, we sought to explore whether sociodemographic factors (age, IQ, sex, socioeconomic status) impact on the extent of knowledge growth. A further explorative aim was to assess participants’ evaluation of the layout of the website as well as their behavioral intention after the reception, e.g. in terms of help seeking behavior. We hypothesized that after consuming the contents of the website, participants would show a growth in knowledge in the specific domains as well as across all domains, which is sustained at follow-up. Based on previous results [30, 31] we assumed that female participants and participants with a higher socioeconomic status would exhibit a more pronounced knowledge gain.

## Methods

### Participants and Recruitment

34 healthy children and adolescents between 11 and 18 years were included in the study (*M*_age_ = 15.65, *SD*_*age*_ = 2.22), of which *n* = 22 were female. Inclusion criteria required an IQ ≥ 80 based on the CFT-20-R (German version) [35] (*M*_*IQ*_ = 110.71, *SD*_*IQ*_ = 11.74) and sufficient German language skills to understand the instructions, the contents of the website, and the questionnaires. Only participants without a current mental disorder were included. The rationale of this approach is that the website “Corona und Du” [34] is a low-threshold offer that particularly addresses healthy youths rather than children or adolescents with mental disorders which are in need of professional help. Likewise, the inclusion criteria regarding age mirrors the fact that the website specifically addresses older youths which were found to be at a particular high risk of poor mental health outcomes during the pandemic [14].

The absence of a mental disorder was assessed using a standardized semi-structured diagnostic interview (Diagnostic Interview for Mental Disorders for Children and Adolescents, Kinder-DIPS) [36, 37]. In addition to the Kinder-DIPS, all participants completed the Beck Depression Inventory-II [BDI-II; 38] to screen for depressive symptoms, which are very prevalent during the current crisis [13, 14]. As can expected in a non-clinical sample comprising participants without a current mental illness, BDI-II scores were low (*M*_*BDI-II*_ = 2.35, *SD*_*BDI-II*_ = 2.36). One participant was excluded due to substantially increased depressive symptoms (*Sum*_*BDI-II*_ = 26, cut-off for minimal depression: BDI-II score ≥ 9), resulting in the final sample of 34 participants as described above. To assess the socioeconomic status index (ses-index) according to Lampert et al. (2018) [39], sociodemographic information was obtained via a parent-report questionnaire which contained items on the educational level of the child and the parents, parents’ professional qualification, and income. For most participants, a high socioeconomic status was reported (17.6% middle; 79.4% high; 1 missing value).

Participants were recruited via a contact list which contains names of families with healthy youths who had expressed interest to participate in studies within the Department of Child and Adolescent Psychiatry, Psychosomatics and Psychotherapy, Hospital of the Ludwig-Maximilians-University (LMU) Munich. In return for their participation, participants received money or vouchers.

## Materials and Procedure

### Website

**Fig. 1:**
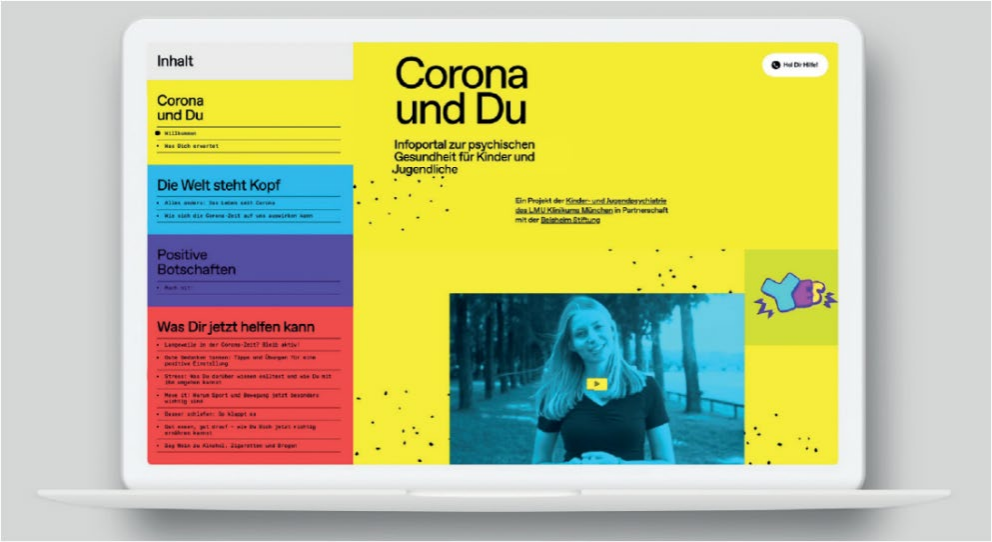
Starting page of the website “Corona und Du”

The web-based information portal “Corona und Du” [34; English translation: “Corona and You”] was launched in Mai 2020 by the Department of Child and Adolescent Psychiatry, Psychosomatics and Psychotherapy of the LMU Hospital Munich together with the “Beisheim Stiftung” (Beisheim Foundation) and media partners (Meiré & Meiré). The website starts off with an introduction section with a short introductory video and an overview of the contents. The website continues with psychoeducation and evidence-based information for children and adolescents as well as advice and supporting information on how to prevent and deal with psychological stress during the corona pandemic (see Fig. 1).

Core contents domains of the website entail (1) “positive thoughts”, (2) “stress management”, (3) “sleep hygiene”, and (4) “nutrition tips”. Furthermore, the website includes links to professional mental health services. In a separate section, the website provides evidence-based information for parents, which was not evaluated in the context of the current study. More details about the development, structure, and dissemination of the website are summarized in the Supplementary Material and [40].

### Assessment of Baseline Knowledge and Knowledge Gain

A self-designed questionnaire was used to assess both the baseline knowledge (pre-assessment; pre), and knowledge gain at post- and follow-up-assessment (post; fu). The knowledge questionnaire included 30 items of which 21 were in a multiple-choice format (with each 4 answer options from which 1 to 4 answers were correct) and nine in a “correct and incorrect” answer format. The items corresponded to the four sections of the website (see Table 1 for item examples). Correct answers were coded as 1, incorrect answers as 0 (sum score range: 0–30; if for one item, several answers options were correct, the answer was only coded as 1 if all answer options were answered correctly). One participant had to be excluded from the analyses on baseline knowledge and knowledge gain due to misunderstanding the instructions (the participant did not understand that the questionnaire included items with several correct answers, resulting in a reduced sample of *n* = 33 for this aspect of the study.

**Table 1:** Example items and answer options of the questionnaire assessing baseline knowledge and knowledge gain

Domain	Number of questions	Example Items	Answer options*
Positive thoughts	6	Making a list or becoming aware of one’s strengths can help to gain confidence and can contribute to a positive attitude.	**(a) right**; (b) wrong.
Stress management	8	Fixed structures in everyday life against stress - what can they look like?	**(a) you can make a schedule to create a fixed structure; (b) you can try to eat and go to bed at about the same time;** (c) you can fill up your schedule to the top. The fewer breaks, the better; **(d) you can plan fixed leisure activities (e.g., inline skating on Wednesday afternoons).**
Sleep hygiene	9	Anna has been lying in bed for about 15 min and can’t fall asleep. What should she do?	(a) Anna should stay in bed and keep trying to fall asleep; **(b) Anna should get up. She should go back to bed when she feels tired**; (c) Anna should pick up her mobile phone and distract herself so that she becomes more tired; (d) Anna should get up and not go back to bed until after midnight, as it is usually easier to fall asleep after midnight.
Nutrition tips	7	Anton eats regular meals throughout the day. This may have a positive influence on his mood and thoughts.	**(a) right**; (b) wrong.

* correct answer responses are written in bold

### Reception of the Website, Behavioral Intention, and Social Desirability

The overall reception of the website, its layout, and usefulness, the reception of the introduction video, as well as participants’ behavioral intention (in sense of higher motivation, e.g. regarding help seeking behavior) were assessed via a self-designed evaluation questionnaire. The questionnaire comprised a four-point rating scale (from 0: not accurate to 3: entirely accurate). Items are summarized in Table 3. In addition, the short version of the Visual Aesthetics of Websites Inventory [VisAWI-S; 41] was used to assess the evaluation of the website’s design. Detailed descriptions of the psychometric properties of all measures used can be found in Supplementary Table 1. Participants indicated their response to the four items (e.g., *“The layout appears professionally designed”*) on a seven-point Likert scale (ranging from 1 „do not agree at all“ to 7 “do fully agree”) [41]. For these four items, a mean value is calculated (general aesthetics factor). As a benchmark, an overall rating of 4.5 or higher means that the website is generally experienced as positive [42, 43]. As answers regarding the reception of the website and behavioral intention can be prone to response biases, we applied the Social Desirability Scale-17 [SDS-17; 44] to examine whether social desirability influenced the responses.

### Procedure

All testings took place at the Department of Child and Adolescent Psychiatry, Psychosomatics and Psychotherapy, Hospital of the Ludwig-Maximilians-University (LMU) Munich. After written informed consent/assent was obtained, participants were interviewed with the Kinder-DIPS to exclude participants with a mental health disorder. Next, the CFT-20-R and BDI-II were conducted (T0). Before parts of the website were presented, participants completed knowledge questionnaire (pre). Participants were instructed to carefully attend to the website and to read the text passages in a concentrated manner. They were guided through parts of the website by an experimenter who scrolled through the website to ensure high experimental control. First, participants watched the introduction video. Afterward, they were asked to consume the website´s main contents in the following fixed order with fixed time windows for the reception: “positive thoughts” (time: 4 min.), “stress management” (time: 4 min.), “sleep hygiene” (time: 4 min.), and “nutrition tips” (time: 2 min.). Participants then had 6 additional min. to scroll through the whole website on their own. Thereafter, participants completed the same knowledge questionnaire (post), which they had completed at pre, as well as the evaluation questionnaire, VisAWI-S, and SDS-17. After 2 weeks, participants took part in a follow-up testing which included the knowledge questionnaire.

### Data Analysis

Statistical data analysis was carried out using IBM SPSS Statistics version 26. For all analyses, the significance level was set to *p* = .05 (two-tailed).

For the knowledge items, we calculated unweighted index values [45] for each participant, both across all domains and for every subdomain. This approach is based on the classical test theory assumption of parallel items, meaning all items are equally good indicators of the underlying construct. Hence, we calculated the index values as added up ordinal scores [46, 47]. To assess baseline knowledge, a proportional score of the mean of the index values divided by the highest possible score was calculated for every subdomain as well as for total knowledge. For a similar approach see [31].

Changes in knowledge over time across all domains were analyzed using a repeated-measures ANOVA for the index value calculated across all domains, with the factor time (pre/post/fu) as a within-subject factor. If the sphericity assumption was violated, Greenhouse-Geisser’s correction was applied (Mauchly’s test). To investigate change in knowledge over time for the four different subdomains, a repeated-measures 3 × 4 ANOVAs with time (pre, post, fu) and domain (positive thoughts, stress management, sleep hygiene, nutrition tips) as the within-subject factors was conducted. Due to the focus of the present study on the main effects, only these were followed up by post-hoc comparisons. In the case of significant effects in the repeated-measures ANOVAs, we conducted further post-hoc comparisons, and adjusted the significance level (*p* = .05 two-tailed) according to the Bonferroni-Holm procedure [48]. For all ANOVAs, effect size partial eta square (*η* ^*2*^) was computed. A small effect is defined as *η* ^*2*^ = 0.01, a medium effect as *η* ^*2*^ = 0.06 and a large effect as *η* ^*2*^ = 0.16 [49].

The influence of socioeconomic status, sex, age, and IQ on baseline knowledge was analyzed using a multiple regression with socioeconomic status, sex, age, school type, and IQ as predictors and baseline knowledge as the criterion. Likewise, to analyze the influence of these predictors on changes in knowledge from pre to post, a multiple regression was computed with the above mentioned predictors and the knowledge difference score (post minus pre) as the criterion. As data on the socioeconomic status of one participant were missing, these analyses were calculated with *n* = 32.

For answers in the questionnaires on the reception of the website (via the evaluation questionnaire and VisAWI-S), and the behavioral intention (via the evaluation questionnaire), descriptive statistics were calculated. Correlations were computed between SDS-17 scores and answers of the evaluation questionnaire to investigate whether participants’ social desirability was related to their answers in this domain. For these analyses, no correction for multiple comparisons was conducted, which in this case is the more conservative approach regarding the validity of our results (i.e., significant results would speak against and not in favour of the validity of our results).

### Power Analysis

An a priori power analysis was computed to determine the necessary sample size to test our hypotheses. The analysis was based on a study which explored the effectiveness of a target-group specific psychoeducation booklet to improve mental health (particularly depression) literacy in healthy youths based on a pre-post-follow-up design [31]. Based on repeated-measures ANOVAs, medium to large effect sizes (*η* ^*2*^ = 0.07 - 0.56) were found regarding knowledge gain. Based on the conservative assumption of an effect size *η* ^*2*^ = 0.07, an alpha error of 0.05 and a power of 0.80, N = 23 participants are needed to detect this effect in a repeated-measures ANOVA. Thus, the sample of the present study with N = 34 was sufficiently large to detect the expected effect.

## Results

### Baseline Knowledge About Mental Health Contents

Participants’ total baseline knowledge about mental health information was *M* = 55.66% (*SD* = 3.77) and ranged from 45 to 74% correct answers in the different domains: “sleep hygiene” (*M* = 45.45%, *SD* = 1.53), “nutrition tips” (*M* = 46.32%, *SD* = 1.12), “stress management” (*M* = 61.36%, *SD* = 1.83), and “positive thoughts” (*M* = 74.24%, *SD* = 1.06).

**Fig. 2:**
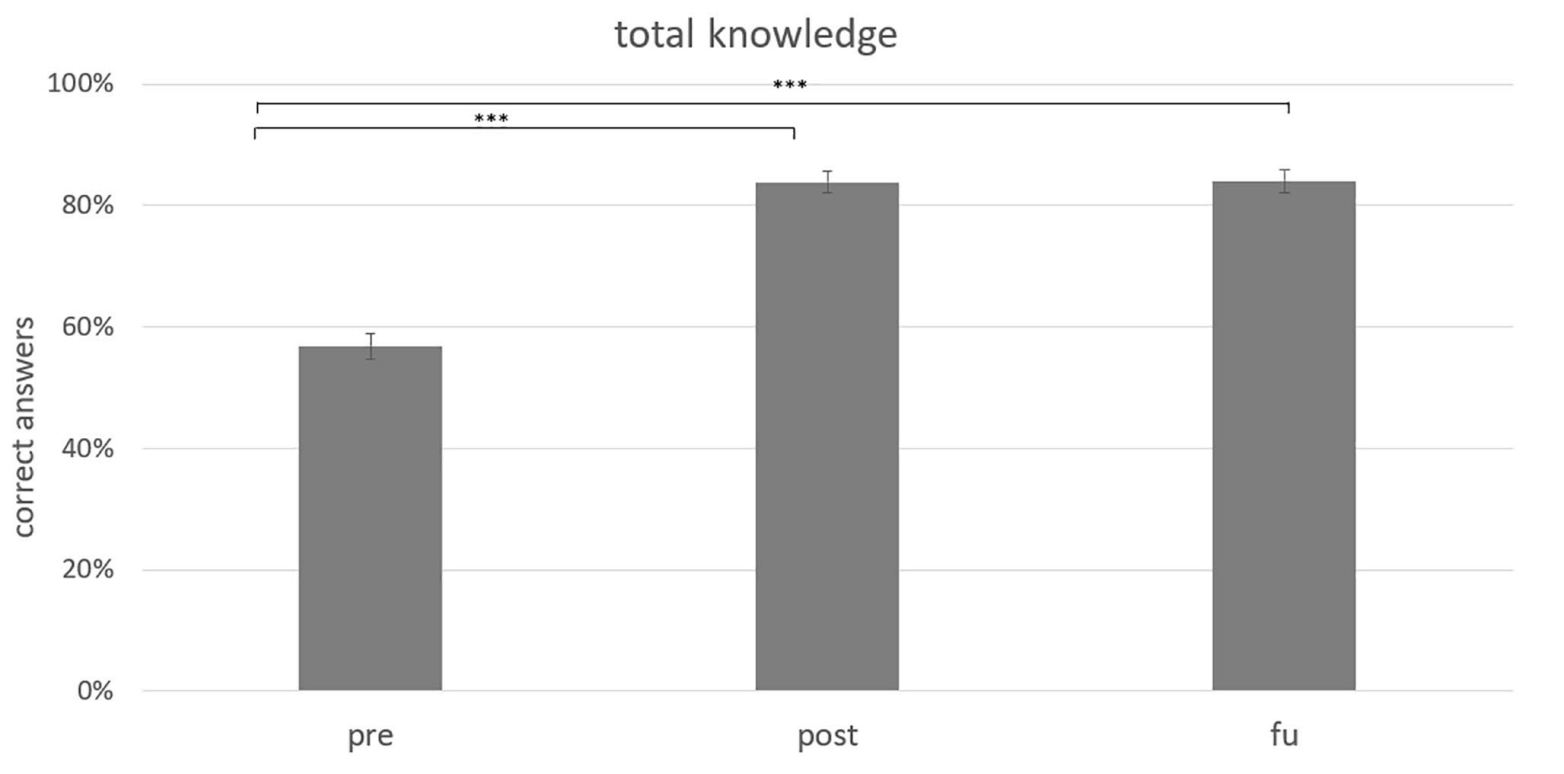
Changes in total knowledge scores over time (in %) ***p < .001 *Note*: pre = pre-assessment. post = post-assessment. fu = follow-up assessment after 2 weeks. Error bars show standard errors

The repeated-measures ANOVA on changes in knowledge across the domains based on the index values revealed a significant main effect of time, *F* (1.60, 51.24) = 136.33, *p* < .001, partial *η²* = 0.81. Post-hoc dependent samples t-tests revealed a significant increase in knowledge from pre to post (*t*(32) = -15.98, *p* < .001) and from pre to fu (*t*(32) = -11.73, *p* < .001). The comparison of index values between post and fu was not significant (*t*(32)= -0.104, *p* = .918) (see Fig. 2).

### Changes in Knowledge Over Time

**Fig. 3:**
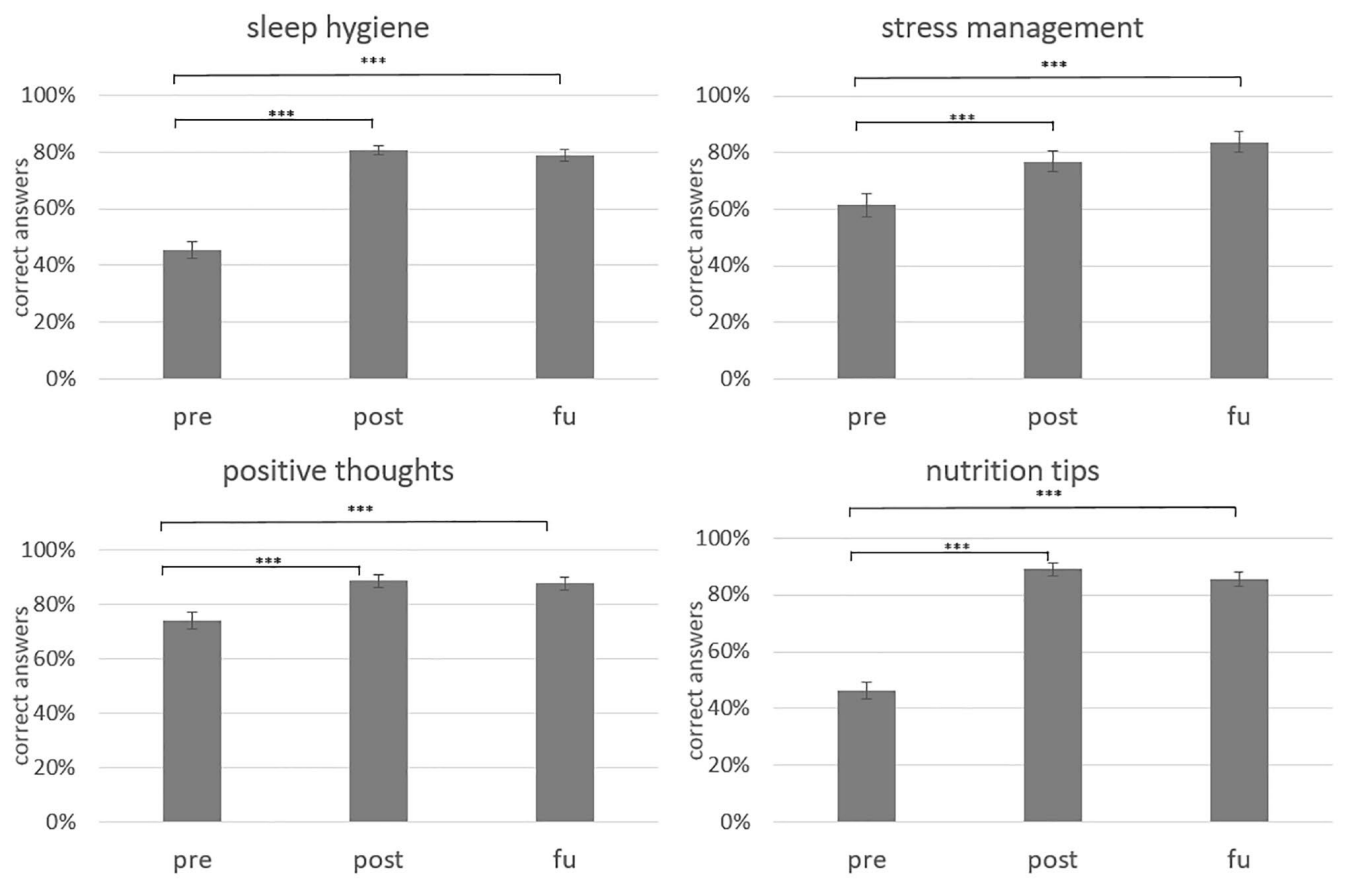
Changes in knowledge over time in the different subdomains (in %) ***p < .001 *Note*: pre = pre-assessment. post = post-assessment. fu = follow-up assessment after 2 weeks. Error bars show standard errors

The repeated-measures ANOVA on changes in knowledge for the four domains revealed a significant main effect of time (*F*(1.6, 51.24) = 136.33, *p* < .001, partial *η²* = 0.810) and domain (*F*(3, 96) = 13.48, *p* < .001, partial *η²* = 0.296). Furthermore, the interaction between time and domains was significant (*F*(6, 192) = 15.06, *p* < .001, partial *η²* = 0.320). Figure 3 presents changes in knowledge over time separately for the four different subdomains. Post-hoc dependent samples t-tests revealed a significant increase in knowledge from pre to post in the subdomains sleep hygiene (*t*(32) = -12.58, *p* < .001), nutrition tips (*t*(32) = -14.37, *p* < .001), stress management (*t*(32) = -4.08, *p* < .001), and positive thoughts (*t*(32) = -5.44, *p* < .001). Moreover, post-hoc tests revealed an increase in knowledge from pre to fu in the subdomains sleep hygiene (*t*(32) = -10.28, *p* < .001), nutrition tips (*t*(32) = -11.38, *p* < .001), stress management (*t*(32) = -5.66, *p* < .001), and positive thoughts (*t*(32) = -4.38, *p* < .001). There was a significant increase in knowledge from post to fu in the subdomain stress management (*t*(32) = -2.09, *p* < .05); however, the difference between post and fu was not significant after Bonferroni correction (see Fig. 3). For the other subdomains, knowledge remained stable from post to fu, i.e., there was no significant difference between post and fu knowledge scores (all *p ≥ * 0.133).

Although the interaction effects were not followed up based on post-hoc t-tests, descriptive statistics suggested that a more pronounced knowledge gain was evident in domains for which baseline knowledge was relatively lower (sleep hygiene, nutrition tips; see Fig. 3).

### Prediction of Baseline Knowledge and Knowledge Changes from Pre to Post

The results of the regression analysis with socioeconomic status, sex, age, and IQ as predictors and baseline knowledge as the dependent variable showed that the variables accounted for a significant proportion of variance (*F*_4,27_ = 7.58; *p* < .001; *R* ^*2*^ = 0.53). While sex, age, and socioeconomic status were significant predictors of baseline knowledge, IQ was not (see Table 2). More specifically, in this model, female participants had a higher baseline knowledge score than male participants. Furthermore, older participants and participants with higher socioeconomic status had a higher baseline knowledge score. The results of the second regression analysis with socioeconomic status, sex, age, and IQ as predictors for knowledge change from pre to post showed that the variables had no significant influence on knowledge gain (F_4,27_ = 1.78; *p* = .162; *R* ^*2*^ = 0.21) (see Table 2). Male participants tended to have a higher knowledge gain (*M* = 9.29, *SD* = 3.05) from pre to post compared to female participants (*M* = 7.43, *SD* = 2.67), albeit this failed to be significant.

**Table 2:** Results of the regression analyses on the prediction of baseline knowledge and knowledge gain from pre to post

		*B*	*SE for B*	*95% CI for B*	*β*	*t*	*p*
***Baseline knowledge***							
Socioeconomic status	2.9		1.4	[0.02, 5.8]	0.31	2.06	0.049
Sex	-3.9		1.1	[-6.2, -1.6]	- 0.51	-3.48	0.002
Age	0.8		0.2	[0.3, 1.3]	0.46	3.47	0.002
IQ	0.04		0.04	[-0.1, 0,1]	0.13	0.89	0.381
***Knowledge gain pre/post***							
Socioeconomic status	-1.6		1.4	[-4.5, 1.3]	- 0.21	-1.1	0.277
Sex	2.04		1.1	[-0.3, 4.4]	0.34	1.8	0.084
Age	-0.1		0.2	[-0.6, 0.4]	- 0.09	-0.5	0.599
IQ	-0.05		0.04	[-0.1, 0.04]	- 0.21	-1.2	0.252

### Reception of the Website and Behavioral Intention

The overall evaluation of the reception of the website ranged between “very good” and “good” (*M* = 1.74 on a rating scale based on the grading system used in German schools from ‘‘very good’’ (1) to ‘‘insufficient’’ (6)). Results of participants’ answers regarding the evaluation of the website as well as behavioral intention are shown in Table 3. In addition, results on the VisAWI-S general aesthetic factor showed that the layout was evaluated as positive (*M* = 5.52, *SD* = 0.20).

**Table 3:** Results for items of the evaluation questionnaire (in %)

Items	entirely accurate	mainly accurate	somewhat accurate		not accurate			
**Reception of the website**								
I would recommend the website to other young people.	44.1	38.2		14.7		2.9		
I think the website is well suited for adolescents.	50.0	44.1		5.9		0		
It was fun to look at the website.	41.2	50.0		8.8		0		
I like the introduction video.	47.1	44.1		8.8		0		
The introduction video provides a good overview of the website.	64.7	29.4		5.9		0		
I like the GIFs on the website.	61.8	29.4		5.9		2.9		
I find the texts interesting.	47.1	44.1		8.8		0		
**Behavioral intention**								
After reading the website, I know better what I can do to feel better in times of the corona pandemic.	50.0	38.2		11.8		0		
After reading the website, I know better whom to turn to in case of psychological problems.	50.0	35.3		11.8		2.9		
Reading the website encouraged me to seek professional help for mental health problems.	35.5	38.2		23.5		2.9		

*Note*: Using a four-point rating scale (0: not accurate, 1: somewhat accurate, 2: mainly accurate, 3: entirely accurate); *%*; N = 34

Correlation analyses revealed that social desirability was unrelated to participants’ response behavior concerning the items of the evaluation questionnaire (all *p* ≥ .068).

## Discussion

The main goal of the study was to investigate whether providing digital information for youths on mental health during the corona pandemic is effective in increasing knowledge in the target group. Moreover, we examined whether sociodemographic factors would impact knowledge increase. In addition, participants’ evaluation of the layout of the website as well as their behavioral intention after receiving the website were assessed.

The results showed increased knowledge across all domains from pre to post and from pre to fu. This increase in knowledge was maintained over the course of two weeks. The extent of knowledge gain was not influenced by demographic variables. Participants‘ evaluation of the layout of the website was positive and their behavioral intention in sense of their motivation to seek help was activated.

Our finding of a stable knowledge gain with large effect sizes supports the effectiveness of our online approach educating children and adolescents on mental health aspects during the corona pandemic. Though evaluation studies on the effects on knowledge gain of mental health websites for children and adolescents in times of COVID-19 are missing, our results can be brought in line with results on the effectiveness of preventive measures for children and adolescents in the field of mental health [50].

Our results on the reported increased behavioral intention underline that the approach taken reduced barriers regarding children and adolescents’ help seeking behavior. Similar digital approaches with children and young people [33] and adults [28] could also show that barriers or help seeking behavior were reduced and higher behavioral intention was found after the reception of a website. It should be pointed out that it was beyond our experimental study to assess whether children and adolescents long-term really sought more professional help after study participation, which would have required a larger sample size. This having said, reports of mental health campaigns show that these approaches impact young people’s awareness regarding mental health literacy, and influence their help seeking behavior [51, 52]. Thus, based on these findings, approaches like ours might help to facilitate children´s and adolescents’ access to the healthcare system and minimize undertreatment.

Our findings suggests that a wide range of children with different cognitive skills and from different backgrounds might profit from the evidence-based information provided, suggesting that our approach can broadly be applied. It should be noted, however, that most of the participating children and adolescents had a high socioeconomic status and an IQ in the above average level. Further research to replicate our results in a more representative sample is therefore necessary. Related to the latter issue, the finding that higher socioeconomic status was associated with higher baseline knowledge can be brought in line with similar findings from former studies on the effectiveness of mental health promotion in children and adolescents [30, 31]. Together, these results suggest that especially disadvantaged groups who were more burdened and affected by increased mental health problems during the pandemic need to be addressed in approaches like ours to close knowledge gaps [13, 15].

The positive evaluation of the design and layout suggests that the website was appealing to young people, which is an important basis to ensure high acceptance and access to the website. Thus, our approach to involve the target groups and to work together with media partners to design an appealing web-based information platform on mental health for children and adolescents proved to be successful. This is also mirrored by the fact that the information portal has achieved a great coverage with > 83.469 visitors and > 165.439 website views in the time period from its launch, May 12, 2020 to August 20, 2022.

## Limitations

There are several study limitations. Since participants’ socioeconomic status was high, the generalizability to other groups with lower socioeconomic status is pending. Yet, especially children and adolescents with low socioeconomic status are at risk of developing mental health problems [53]. It is therefore important to particularly reach out for these groups to prevent adversity. The interpretation of our results is limited by the fact that the design of the study was not randomized-controlled. Thus, to be able to draw more stringent conclusions about causality regarding observed knowledge gain, future studies should apply a randomized-controlled study design. Moreover, it was beyond the present study to assess the transfer of the acquired knowledge into real life or proof of implementation of the different tips into everyday life, which both should be addressed in future studies. Although guiding participants through the website with the help of the experimenter allowed high experimental control, one disadvantage is that it remains unknown on how youths naturally view the website. In future studies it would be very worthwhile to follow-up this aspect, e.g., by conducting an eye-tracking study to gain insight which content sections receive most attention.

## Conclusion

In the context of the negative consequences of the pandemic, especially children and adolescents, and particularly those who are already disadvantaged are in urgent need of evidence-based information on mental health promotion. To our knowledge, this study is the first to show that an evidence-based information platform on mental health for children and adolescents during the COVID-19 pandemic is effective and perceived as attractive in enhancing knowledge as well as in changing children and adolescents’ behavioral intentions. Our study provides important implications for the design of future online portals that aim at informing youths about evidence-based strategies to maintain their mental health in times of crisis and beyond. Importantly, target-group specific online portals like “Corona und Du” [34] offer the possibility to reach children and adolescents regardless of place, time and financial resources. Such offers represent a low-threshold approach to reduce bottlenecks in health care and thus contribute to the equity of the health care system [54, 55].

## Summary

With the beginning of the corona pandemic, especially children and adolescents were confronted with substantial challenges in their everyday life and thus in urgent need of information on how to maintain their mental health. To address this need, we developed a web-based information platform on mental health for children and adolescents (www.corona-und-du.info), which contains evidence-based information, advice, and hands-on-support on how to prevent and deal with psychological stress during the corona pandemic. This present experimental pre-post-follow-up study examined whether the consumption of the website´s mental health contents lead to knowledge gain in the target group. Moreover, the reception of the website and changes in the behavioral intention to seek psychological help were investigated. Thirty-four healthy children and adolescents aged 11–18 years participated in the study. Participants were presented content subdomains of the website in the laboratory setting. Before (pre) and after (post) consumption of the contents, as well as at a two-week follow-up, we applied a 30-item questionnaire to assess knowledge gain. Furthermore, participants evaluated the reception of the website and changes in their behavioral intention to seek help based on standardized questionaries. For all content domains investigated, we found a significant knowledge gain with large effect sizes from pre to post, as well as for pre to follow-up. The overall reception of the website was positive (*M* = 1.74 on a rating scale from “1-very good” to “6-insufficient”), as was the reception of the website´s design (*M* = 5.52, *SD* = 0.20 on scale ranging from 1 “don´t agree at all“ to 7 “fully agree”). Moreover, participants indicated increased behavioral intention to seek psychological support when needed. The results indicate that the approach taken is efficient and appealing in enhancing knowledge on mental health in youths and has the potential to reduce barriers with respect to help-seeking behavior. The results constitute an important basis for future attempts to promote mental health information in children and adolescents in challenging times like the corona pandemic.

### Electronic supplementary material

Supplementary table for Table 3 (DOC 38 kb)
