# Supplementary material for: Evaluation of a Web-based Information Platform for Youths on Mental Health During the COVID-19 Pandemic
Source: Child Psychiatry Hum Dev. 2022 Oct 20;55(3):861–71. doi: 10.1007/s10578-022-01425-5 (PMC9583065; doi:10.1007/s10578-022-01425-5)
Supplement: Supplementary file 1 — Supplementary table for Table 3 (DOC 38 kb) [file 10578_2022_1425_MOESM1_ESM.doc]

# Supplementary Material

**Development, structure, and dissemination of the website**

The content domains were selected based on an extensive literature search on effective mental health promotion for children and adolescents [1-5] as well as on effective strategies for the prevention of depression in youths [6]. For each of these domains, the website contains different tips and recommendations for the target group, thereby referring to the current challenges of the pandemic situation. For instance, within the domain “positive thoughts”, we provide tips like encouraging children and adolescents to reflect on their strengths in light of the current crisis. The domain “stress management” provides - among other aspects - recommendations on how to maintain structure in everyday life when confronted with countermeasures such as school closures (e.g., with the help of a weekly schedule) and information on relaxation exercises. The domain “sleep hygiene” refers to tips for the ideal place to sleep (e.g., without electronic devices) or tips on sleeping times (e.g., with fixed sleeping times). The component “nutrition tips” contains recommendations for healthy eating. All tips can be easily implemented in everyday life and are intended to strengthen the self-efficacy of children and adolescents in times of the corona crisis.

The contents, language, and layout of the information portal have been created with the help of interviews with children and adolescents to appeal to the target group. The texts are easy to understand and are written in a motivating tonality. The website contains GIFs, which intend to underline the content. The conception and design of the website, as well as the technical development, were commissioned by a professional design agency. The dissemination was primarily carried out through network communication by addressing relevant stakeholders (such as ministries of health and educational as well as health institutions and practioners) as well as through the press [for details see 7].

# Supplementary Tables

**Supplementary Table 1.** Psychometric information about the measures used

| Measure | Instrument | Reliability/validity |
| --- | --- | --- |
| Diagnostic interview | Kinder-DIPS | The DSM IV version of the interview has good retest and interrater reliabilities (Cohens Kappa ≥ 0.90) and has shown good validity [8, 9]. |
| IQ test | CFT-20-R | The instrument has excellent reliability (Cronbach’s α = 0.96) and correlates well with other IQ tests [10]. |
| Depressive symptoms | BDI-II | In our sample, internal consistency was excellent (Cronbach’s α = .903). Based on a meta-analysis [11] the pooled estimate for internal reliability is 0.86 and the discriminative validity pooled estimate for sensitivity and specificity is 0.81. |
| Knowledge | Self-designed knowledge questionnaire | In our sample, the internal consistency (Cronbach’s α) for the total knowledge score was 0.623 (pre), 0.718 (post), and 0.766 (follow-up), respectively. |
| Reception of the website and behavioral intention | Self-designed evaluation questionnaire | In our sample, internal consistency was 0.82 (Cronbach’s α). |
| Reception of the website | VisAWI-S | The instrument has shown convergent and divergent validity and high internal consistency with Cronbach’s α = 0.81. [12]. In our sample, internal consistency was 0.81 (Cronbach’s α). |
| Social desirability | SDS-17 | The instrument shows satisfactory internal consistency (Cronbach’s α = 0.72 – 0.75) and substantial validity (convergent validity and discriminant validity) [13, 14]. In our sample, internal consistency was 0.73 (Cronbach’s α). |

*Note.* BDI-II = Beck Depression Inventory-II. CFT-20-R = Culture Fair Intelligence Test-Revised. Kinder-DIPS = Diagnostisches Interview bei psychischen Störungen im Kindes- und Jugendalter [diagnostic interview for mental disorders in childhood and adolescence]. SDS-17 = The Social Desirability Scale-17. VisAWI-S = Visual Aesthetics of Websites Inventory.

**Supplementary References**

1. Cairns KE, Yap MBH, Pilkington PD, Jorm AF (2014) Risk and protective factors for depression that adolescents can modify: a systematic review and meta-analysis of longitudinal studies. J Affect Disord 169: 61-75

2. Firth J, Solmi M, Wootton RE, Vancampfort D, Schuch FB, Hoare E, et al (2020) A meta‐review of “lifestyle psychiatry”: the role of exercise, smoking, diet and sleep in the prevention and treatment of mental disorders. World Psychiatry 19: 360-380

3. Loewen OK, Maximova K, Ekwaru JP, Faught EL, Asbridge M, Ohinmaa A, et al (2019) Lifestyle behavior and mental health in early adolescence. Pediatrics 143: e20183307

4. Catalano RF, Fagan AA, Gavin LE, Greenberg MT, Irwin Jr CE, Ross DA, et al (2012) Worldwide application of prevention science in adolescent health. The Lancet 379: 1653-1664

5. Saxena S, Jané-Llopis E, Hosman C (2006) Prevention of mental and behavioural disorders: implications for policy and practice. World Psychiatry 5: 5-14

6. Cairns KE, Yap MB, Reavley NJ, Jorm AF (2015) Identifying prevention strategies for adolescents to reduce their risk of depression: a Delphi consensus study. J Affect Disord 183: 229-238

7. Piechaczek CE, Primbs R, Comanns P-M, Feldmann L, Greimel E, Schulte-Körne G (2021) Corona und Du: ein webbasiertes Infoportal zur psychischen Gesundheit für Kinder, Jugendliche und Eltern. Nervenheilkunde 40: 333-340

8. Adornetto C, In-Albon T, Schneider S (2008) Diagnostik im Kindes- und Jugendalter anhand strukturierter Interviews: Anwendung und Durchführung des Kinder-DIPS. Klin Diagnostik u Evaluation 1: 363-377

9. Neuschwander M, In-Albon T, Adornetto C, Roth B, Schneider S (2013) Interrater-Reliabilität des Diagnostischen Interviews bei psychischen Störungen im Kindes- und Jugendalter (Kinder-DIPS). Z Kinder Jugendpsychiatr Psychother 41: 319-334

10. Weiß RH (2019) CFT 20-R, Grundintelligenztest Skala 2 – Revision, 2nd edn. Hogrefe, Göttingen

11. Stockings E, Degenhardt L, Lee YY, Mihalopoulos C, Liu A, Hobbs M*,* et al (2015) Symptom screening scales for detecting major depressive disorder in children and adolescents: a systematic review and meta-analysis of reliability, validity and diagnostic utility. J Affect Disord 174: 447-463

12. Moshagen M, Thielsch M (2013) A short version of the visual aesthetics of websites inventory. Behav Inf Technol 32: 1305-1311

13. Stöber J (2001) The Social Desirability Scale-17 (SDS-17): convergent validity, discriminant validity, and relationship with age. Eur J Psychol Assess 17: 222-232

14. Stöber J (1999) Die Soziale-Erwünschtheits-Skala-17 (SES-17): Entwicklung und erste Befunde zu Reliabilität und Validität [The social desirability scale-17 (SDS-17): development and first findings on reliability and validity]. Diagnostica 45: 173-177
